# Influence of Emulsifiers and Dairy Ingredients on Manufacturing, Microstructure, and Physical Properties of Butter

**DOI:** 10.3390/foods10051140

**Published:** 2021-05-20

**Authors:** Bhavesh Panchal, Tuyen Truong, Sangeeta Prakash, Nidhi Bansal, Bhesh Bhandari

**Affiliations:** 1 ARC Dairy Innovation Hub, School of Agriculture and Food Sciences, University of Queensland, Brisbane, QLD 4072, Australia; b.panchal@uq.edu.au (B.P.); s.prakash@uq.edu.au (S.P.); n.bansal@uq.edu.au (N.B.); 2 School of Science, RMIT University, Melbourne, VIC 3028, Australia; tuyen.truong@rmit.edu.au

**Keywords:** emulsifiers, dairy ingredients, butter, microstructure, texture, rheology, tribology, functionality

## Abstract

The influence of emulsifiers and dairy solids on churning and physical attributes of butter was investigated. Commercial dairy cream was blended with each of the ingredients (0.5%, *w*/*w*) separately, aged overnight (10 °C), and churned (10 °C) into butter. The employed additives showed a distinctive impact on the macroscopic properties of butter without largely affecting the melting behavior. In fresh butter, polyglycerol polyricinoleate (PGPR) emulsifier having dominated hydrophobic moieties significantly (*p* < 0.05) enhanced the softness. Among dairy solids, sodium caseinate (SC) was the most effective in reducing the solid fat fraction, hardness, and elastic modulus (G’), while whey protein isolate (WPI) and whole milk powder (WMP) produced significantly harder, stiffer, and more adhesive butter texture. As per tribological analysis, PGPR, Tween 80, and SC lowered the friction-coefficient of butter, indicating an improved lubrication property of the microstructure. The extent of butter-setting during 28 days of storage (5 °C) varied among the samples, and in specific, appeared to be delayed in presence of WPI, WMP, and buttermilk solids. The findings of the study highlighted the potential of using applied emulsifiers and dairy-derived ingredients in modifying the physical functionality of butter and butter-like churned emulsions in addition to a conventional cream-ageing process.

## 1. Introduction

Butter is a milk fat-based product generally obtained through phase inversion of partially crystallized cream by means of mechanical churning and working. Cream is an oil-in-water type emulsion containing milk fat in the form of globules. Fat globules are stabilized by a complex interfacial membrane and exist in wide size range from about 0.1–15 μm [1]. Partial crystallization of fat is crucial for the disruption of fat globule membrane and partial coalescence of the fat globules during churning. The leakage of liquid fat during the process enables the formation of fat clumps, which continue to grow in size until the fat phase (butter grains) and serum phase (buttermilk) become visibly separate [1,2]. After separation of buttermilk, butter grains are worked to form a uniform water-in-oil type viscoelastic emulsion. The microstructure of final butter product comprises continuous liquid fat phase, fat crystal-network, dispersed water phase, denuded and intact fat globules, air, residual milk fat globule membrane, and other non-fat solids [3,4].

Texture is one of the most important quality parameters determining the spreadability, sensory properties, and consumer acceptability of butter. Many different approaches have been exploited to achieve desired textural quality of butter, which includes temperature treatment of cream [5,6,7], churning and working conditions [8,9], inclusion of low melting milk fat fraction [10,11], specified cow diet [12,13,14], and CO_2_-aided crystallization [15]. In addition, size and interfacial components of the fat globules were also observed to impact the churning efficiency [16] and physical functionality of resultant butter [17]. Fat globules of controlled sub-micron sizes emulsified with sodium caseinate (SC) and Tween 80 were found to enhance the softness of butter, whereas that with only SC had exhibited a harder texture [17]. Therefore, other than fat globule size, additives (SC and Tween 80) played an interactive role in governing the textural properties of butter. The effects of food additives mixed into cream or directly added to butter were studied in the past by Kapsalis, Kristoffersen, Gould, and Betscher [18]. They noticed that incorporation of surfactants (tweens, spans, lecithin, glycerides) and buttermilk solids individually or in combination could improve the softness of butter by 10–44%. Similarly, Wright et al. [9] reported that addition of 1% (*w*/*w*) monoglycerides in the cream before churning can produce a softer butter. The observed effects were attributed to the emulsifying properties of the used additives, which nevertheless tended to be neutralized over the storage as the setting of butter was delayed for a certain duration but not prevented entirely. Presence of non-fat components and emulsifier additives in bulk fat or emulsions has been known to induce a heterogeneous nucleation process and a mechanism of secondary nucleation [19,20,21]. Besides, depending on the molecular structures and mobility, emulsifiers at the water/oil interface can affect the droplet-matrix interactions, and in consequence, mechanical properties of the product, which has been examined in water-in-oil emulsions [17,22,23] and protein gels [24,25]. However, the role of dairy-derived ingredients has not been explored much in the same light for their impact on the macroscopic consistency of butter. Major products in the dairy ingredients portfolio are dried powders, which are not only used for recombination or reconstitution but have broader applicability. Dairy powders have been used as food ingredients for their intrinsic functional properties in dairy, confectionery, bakery, and meat products [26,27].

The present study was aimed to investigate the effects of series of different dairy solids viz; SC, whey proteins (whey protein isolate; WPI), milk protein concentrate (MPC), buttermilk powder (BMP), and whole milk powder (WMP) on physical properties of butter. Additionally, Tween 80 and polyglycerol polyricinoleate (PGPR) were also employed as relatively hydrophilic and hydrophobic emulsifiers, respectively. As per regulatory standards, butter shall not contain any foreign emulsifying ingredients [28], hence to clarify that the product discussed as butter in this work is not in regulatory terms but based on the churning methodology employed and to interpret the effects of additives. Each additive was blended separately into the cream prior to aging and churning steps. A laboratory-scale batch churning process was used to prepare the butter. The final butter was examined for textural and viscoelastic properties, melting behavior, tribological pattern, and microstructure during 28 days of storage (5 °C). The understanding of the technological functionality of emulsifiers and dairy ingredients would be useful in tailoring the physical functionality of milk fat-based systems like dairy spreads and butter-like churned emulsions.

## 2. Materials and Methods

### 2.1. Materials

#### 2.1.1. Dairy Cream

Commercial dairy cream (Parmalat Australia Pty Ltd., Brisbane, Queensland, Australia) (39.4% *w*/*v* fat) was used as source cream for butter making. According to the manufacturer, it was originated solely from milk and did not contain any additives. The procured cream packs were ensured to be from the same batch and stored at 4 °C until the use. Each batch of the cream was analyzed for fat content (40.1 ± 0.1%, *w*/*w*) to ensure that there is no batch-to-batch variation.

#### 2.1.2. Additives

Dairy ingredients; sodium caseinate (SC: 92.6% protein, 0.25% lactose-casein, 0.7% fat, 1.2% sodium, Murray Goulburn Co-op, Melbourne, Victoria, Australia), milk protein concentrate (MPC: 81.5% protein, 1.3% fat, 5% lactose; Tatura milk industries Pty. Ltd., Tatura, Victoria, Australia), whey protein isolate (WPI: 90.4% protein, 1.0% fat, 0.9% lactose; Fonterra Australia Pty Ltd., Darnum, Victoria, Australia), buttermilk powder (BMP: 31.0% protein, 7.8% fat, 50% carbohydrate; Fonterra Australia Pty Ltd., Darnum, Victoria, Australia) and whole milk powder (WMP: 40.3% lactose, 26.3% fat, 24.5% protein; Fonterra Australia Pty Ltd., Darnum, Victoria, Australia), and emulsifiers; Tween 80 (Tween 80; polyoxyethylene sorbitan monooleate C64H124O26; Labtek Pty Ltd., Brendale, Queensland, Australia, HLB = 15) and polyglycerol polyricinoleate (PGPR, Danisco, Penang, Malaysia, HLB ~ 3) were employed as additives.

### 2.2. Methods

#### 2.2.1. Preparation of Cream Samples

Each of the additives was added separately into the cream. The concentrations of fat and additive were adjusted to 38% (*w*/*w*) and 0.5% (*w*/*w*), respectively, by adding water. The samples were then warmed to 45 °C for 1 h under continuous low speed stirring (200 rpm) to enable proper blend of additives and melting of the fat. After that, the samples were kept in a refrigerated incubator and stored for 15–17 h for isothermal (10 °C) aging.

#### 2.2.2. Butter-Making

After the aging treatment, cream samples were subjected to butter-making using a batch churning method [17]. The developed method employed a UMC-5 Stephan mixer (Stephan Machinery GMBH, Hameln, Germany), having a double-jacketed mixer bowl (5 L capacity) and a temperature controlling unit (Julabo GmbH, Seelbach, Germany). Temperature control mechanism included circulation of conditioned water through the jacket of mixing bowl. The cream sample was poured into a mixer bowl (set at 10 °C), and churning was carried out through high speed (3000 rpm) rotating four-wing blade. Completion of churning was observed as separation of two phases, the aggregated butter grains and separated buttermilk. The required duration of mixing until the phase inversion was recorded as the churning time. The buttermilk was separated out through a screen filter. The retentate butter grains were then evenly kneaded against the Stephan mixer bowl wall (10 °C) to squeeze out the loosely bound serum phase. After that, the consistent weight of butter sample was packed into 70 mL PS containers. A total of seven additives were used in this study, so seven butter samples were made using each additive. A control butter was also prepared under similar process conditions without any extraneous ingredient. In total, eight butter samples were prepared in duplicate and subjected to various analyses during the 28 days of storage (5 °C).

### 2.3. Analyses

#### 2.3.1. Fat and Moisture Content

The fat proportion in the cream was quantified using the Gerber test method as described in BIS Standard 1224-II [29]. The homogenous cream sample was weighed to 5 g in the glass beaker of cream butyrometer. The beaker was then plugged into the butyrometer (50% scale). Ten mL volumes of sulfuric acid (90–91% by mass, density-1.807–1.812 g mL^−1^ at 27 °C) and distilled water each were transferred successively into the butyrometer. The neck of the butyrometer was closed with rubber stopper, and content was mixed thoroughly to dissolve the curd particles. One mL of amyl alcohol was added, followed by a few mL of distilled water to adjust the level of content to 40% mark. The butyrometer was then inverted a few times (to mix the content) and quickly transferred to water bath, keeping bulb uppermost at 65 ± 2 °C for at least 5 min. Butyrometers were then placed into Gerber centrifuge, balancing the rotating disc and centrifuged at maximum speed (1300 rpm) for 5 min. Butyrometers were again transferred into a water bath at 65 ± 2 °C for a minimum of 3 min, and a reading of the fat column was taken. Each sample was analyzed in duplicate.

The moisture content of butter samples was determined through heat desiccation method as described in AOAC method 920.116 [30]. The butter sample was weighed to 3 g in a moisture dish and placed in an oven at 102 °C for a minimum of 2 h to allow all the water to evaporate and thereafter placed in a desiccator for 30 min at room temperature. The moisture was calculated as the percentage difference in the weight of the sample before and after evaporation. The measurement was carried out in duplicate for each sample.

#### 2.3.2. Microstructure

Microstructure of butter was examined through a confocal laser scanning microscope (Zeiss LSM 700, Oberkochen, Germany) using a 63× water-immersion objective. The butter sample was cut into a thin layer of ~1 mm thickness and 1 cm diameter using a metal borer and wire cuter. Two staining agents, namely, Fluorescein Isothiocyanate Isomer I (FITC) and Nile Red, both separately dissolved to 0.01% (*w*/*v*) concentration in acetone were employed. Butter slice was kept on a microscopic slide and a 100 µL volume of Nile red dye was first added onto the sample. After evaporation of the acetone, 100 µL FITC was added. The complete sample preparation was carried out in cold room (~5 °C). The sample was then allowed to equilibrate at 5 °C for minimum 30 min before conducting the analysis. Argon/ArKr 488 nm laser was used for excitation to induce fluorescence emission.

#### 2.3.3. Thermal Property

The melting behavior of butter samples was analyzed using a differential scanning calorimeter (DSC1, Mettler- Toledo, Schwerzenbach, Switzerland). DSC was first calibrated with an indium standard (melting point 156.66 °C, ΔH melting 28.41 J g^−1^). The butter sample was weighed to around 8–12 mg into a 40 μL aluminum pan and sealed hermetically. The sample preparation was carried out in cold room at 5 °C. An empty sealed pan was placed as a reference in DSC chamber. After that, a temperature ramp from 5 °C to 60 °C was carried out at 5 °C min^−1^ heating rate. STARe Excellence Software (Mettler- Toledo, Schwerzenbach, Switzerland) was used to analyze the thermographs for peak melting temperatures. Based on total area of the enthalpy curve, proportion of solid fat in butter was estimated [31]. Due to the varying level of water content in the butter samples, the solid fat fraction was reported on a dry basis.

#### 2.3.4. Texture Analysis

The textural attributes of butter were evaluated using a TA.TX plus texture analyzer (Stable Micro Systems, Surrey, UK). An empirical test based on puncture penetration method was followed using a 4 mm diameter cylindrical probe [17]. The maximum penetration force (N) required for the probe to travel a distance of 10 mm in a sample (50 mm height × 44 mm diameter) with a test speed of 1 mm s^−1^ was recorded as relative hardness. The maximum negative force during the backward movement of the geometry to starting position was noted as adhesiveness. The total time required for each measurement was 30 s maximum. The test was carried out at room temperature (22.5 ± 1.5 °C), and the samples were kept at refrigeration temperature (5 °C) until analyzed. For each replicate, at least four samples were analyzed to record four measurements.

#### 2.3.5. Rheology

The viscoelastic property of butter was characterized using the AR G-2 Rheometer (TA Instruments, New Castle, DE, USA) with a parallel plate (40 mm dia) fixture. The lower stage was equipped with Peltier element to control the temperature. Both the plate geometry and Peltier stage were covered with fine sandpaper (120 grit) to prevent the product from slipping during the oscillatory movement of geometry plate [32]. The butter sample was (5 °C) cut into circular discs (35 mm diameter and 4 mm height) using a pre-cooled stainless steel cylindrical borer and a wire-cutter. A disc was placed on a Peltier stage (temperature set at 5 °C), and then the upper geometry plate was lowered to 4 mm gap. A prerequisite normal force of 10 N was also set to hold the sample firmly between the plates [33]. Thereafter, a frequency sweep test was performed, varying the oscillation frequency from 0.5–500 rad s^−1^ and keeping the oscillation stress consistent at 500 Pa (within linear viscoelastic region). Each replicate was analyzed for a minimum of four measurements using separate butter discs.

#### 2.3.6. Tribology

The lubrication property of butter was evaluated using a Discovery Hybrid Rheometer (TA Instrument, USA) with 3-ball geometry and cup plate assembly. The cup plate was surfaced with transpore tape (3M Health Care, Saint Paul, MN, USA) to ensure proper hold of the sample. Butter sample (5 °C) cut in the form of disc (35 mm diameter and 2 mm thickness) was placed in the cup plate, and upper 3-ball plate was lowered close to the surface of sample. The cup plate was connected to a Peltier stage for control of the temperature. A measurement was performed at 15,000 μm s^−1^ rate of sliding and 2 N of ageometry axial force during a temperature ramp from 5 °C to 35 °C at 5 °C min^−1^. A minimum of two measurements (using separate discs) were carried out for each replicate. After each test, a new piece of transpore tape was layered on the cup plate.

#### 2.3.7. Statistical Analysis

As mentioned previously, butter samples were prepared in two independent replicates, and each replicate was analyzed for at least either two or four measurements depending on the variability in respective analyses. The data was analyzed using MINITAB^®^ 17 (Minitab Co., State College, PA, USA) statistical package, employing Tukey’s multiple comparison test. One-way analysis of variance (ANOVA) was used to determine the significant differences in treatment means at *p* < 0.05.

## 3. Results and Discussion

### 3.1. Butter-Making: Churning Time and Moisture Content

At similar set of experimental conditions, the presence of PGPR, and dairy ingredients; BMP, WMP, WPI, and MPC, exhibited non-significant changes in churning time as compared to the control, whereas SC and Tween 80 produced an evident impact (Table 1). To note, in the presence of SC, time required for phase inversion was prolonged to 315 s, which was notably (*p* < 0.05) higher than the control cream. Contrarily, Tween 80 shortened the churning time by 29% (*p* > 0.05) to 160 s. The observed effects are in agreement with previous findings where conformational changes in fat globule membrane due to adsorption of Tween 80 and SC were linked [16]. Caseinates form a thick viscoelastic interfacial membrane on the fat droplets [34], which can protect the fat globules against the shearing forces and crystal protrusion, leading to better physical stability [1]. Whey proteins also adsorb to interface through formation of interconnected viscoelastic film and exhibit comparable emulsifying properties to that of caseinates [35,36]. However, due to differences in the molecular structures, two types of proteins form structurally different adsorption layers that impact the mechanical strength and rheological properties of the interface [37,38,39]. Based on a physicochemical factors, such as, at pH higher than the isoelectric point (pI = 4.6), a flexible structure of sodium caseinate unfolds quickly at the interface that forms thicker (≈10 nm) and denser interfacial film, whereas globular structures of whey protein could not form a sufficiently compact layer (≈2 nm) to ensure better stabilization [40,41]. Hence, unlike SC, the churning duration of WPI-added cream was not significantly changed compared to control. On the other side, small molecular surfactant Tween 80, being more surface-active, hinders protein interactions on fat globule interface during competitive adsorption, causing an increased probability of rupture of the globule membrane during the churning process [42,43]. Depending on the surfactant to protein molar ratio, complete or partial displacement of protein could happen on the globule surface [42,44]. However, the destabilization of interface is not always due to competitive displacement of protein as such, but perhaps a consequence of coexistence of both proteins and surfactant adsorbed to the surface [44].

The proportion of moisture in all additive containing butter samples, except PGPR butter, was in the range of 14.91–16.89% (*w*/*w*) (Table 1), which showed a minor difference as compared to the control (15.47%, *w*/*w*) and was approximate to the regulatory requirement of 16% (*w*/*w*) maximum [28]. PGPR butter contained a relatively higher amount of water phase (21.50 ± 1.19%, *w*/*w*), where the difference with BMP, WMP, WPI, and Tween 80 butter was significant. The observed effect is conceivably the result of an excellent emulsifying property of PGPR that can aid in stabilization and retention of water in the free fat phase [45] during churning and working processes. Nevertheless, the moisture level observed was comparable to that reported (21–28%) elsewhere [7,15] in butter made with a laboratory-scale setup.

### 3.2. Microstructure

**Figure 1:**
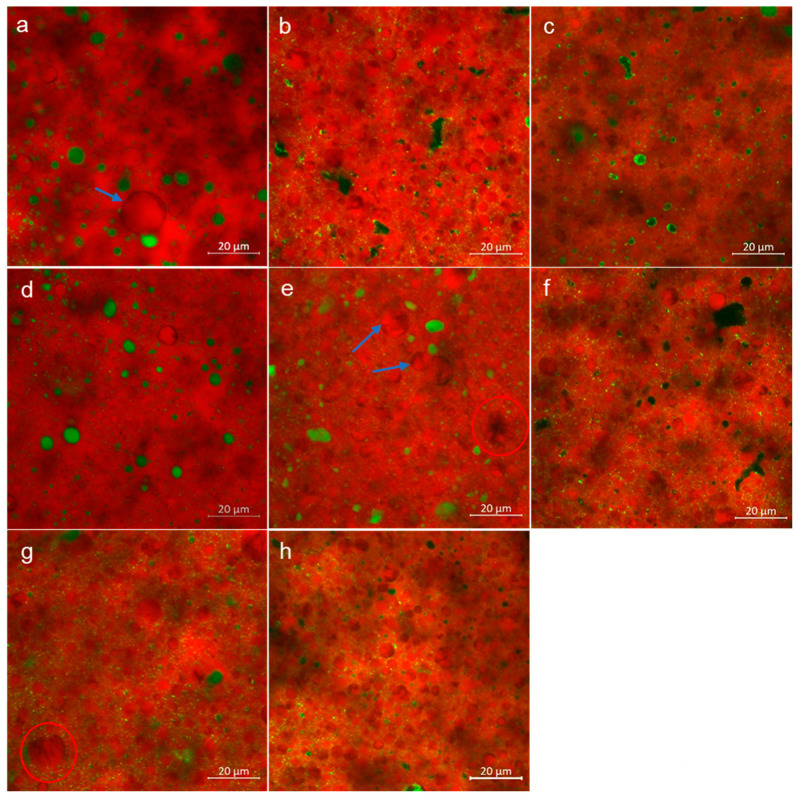
Confocal laser scanning microscopy (CLSM) images of control (without any added additives) (**a**), PGPR: polyglycerol polyricinoleate (**b**), Tween 80 (**c**), SC: sodium caseinate (**d**), BMP: buttermilk powder (**e**), WPI: why protein isolate (**f**), MPC: milk protein concentrate (**g**), and WMP: whole milk powder (**h**) butter. The fat phase (red) is labeled with Nile Red and water phase (green) with fluorescein isothiocyanate (FITC). The dark shadows are network of fat crystals. Blue arrows indicate presence of fat globules. Red circles highlight the crystal aggregates formed within denuded globules. Image (**a**) of control reused from Panchal, et al., 2021 [17]. Copyright 2021 Elsevier.

**Figure 2:**
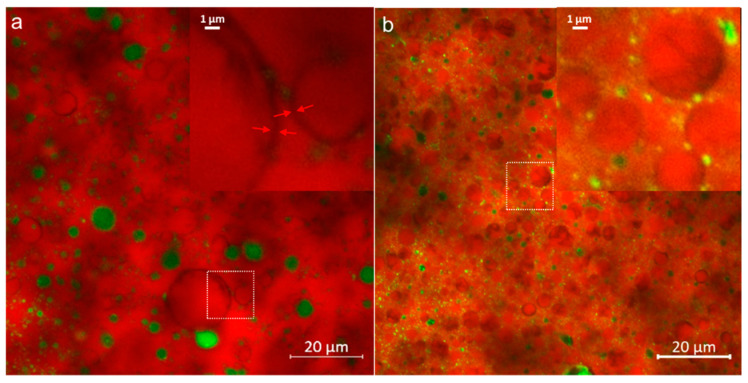
Insert image to highlight (**a**) peripheral crystal fat layer in intact or partially denuded globules, and (**b**) yellow to green colored tiny aqueous droplets dispersed in interglobular spaces in continuous fat phase of butter. Image (**a**) reused from Panchal, et al., 2021 [17]. Copyright 2021 Elsevier.

A typical butter microstructure was observed through CLSM analysis, as could be seen in representative images in Figure 1. Liquid fat in the bright red color was seen as continuous phase with dispersed aqueous phase in green. Distribution of water in the form of droplets was seen up to ~10 µm size, comparable to that reported for butter made through batch-type churning [1], and few irregularly shaped pockets were seen such as in PGPR (b) and WPI (f) butter. The fat crystal network appeared as non-spherical dark shadows as absorption of Nile Red gets limited in the crystalline fat [46]. The dispersed water droplets surrounded by solid fat, therefore, appeared darker as well. The butter matrix also comprises an embedded globular fat phase containing partially disrupted and intact fat globules survived during the churning process, indicated by blue arrows in the images. During aging treatment of cream, crystallization of fat initiates with high melting triacylglycerides that align to the interface of globule [47]. Formation of this peripheral crystalline shell was observed as dark solid fat layer of 0.1–0.5 µm thickness (Figure 2a insert). The fat crystal aggregates and networking was also visible within the intact and partially denuded globules, as highlighted with red circles (Figure 1). Thus, an arrangement of all structural elements forming a multiphase stable microstructure was observed through CLSM. The dispersed aqueous phase consists of serum solids dissolved and suspended in it. The amphiphilic molecules such as proteins and emulsifiers align to the interface due to the affinity of hydrophobic groups for the surrounding fat phase. In PGPR and Tween 80 butter, the arrangement of such surface-active moieties at the water-fat interface was seen as green highlights due to high fluorescent intensity. Similarly, the distribution of proteins in WPI (f), MPC (g), and WMP (h) butter was noticeable as bright yellow to green spots. The distribution of such amphiphiles hydrated with the water layer leads to formation of very minute dispersed droplets of <1 μm size, as was noticeable in the interglobular spaces throughout the structure (Figure 2b insert). Presence of such additive molecules could also form ultra-dispersed water particles of <100 nm size scale at the interfaces between triglyceride monomolecular layers [48].

### 3.3. Physical Properties

#### 3.3.1. Thermal Properties

The comprehensive effect of employed additives on melting characteristics and solid fat content (SFC) of butter was investigated through DSC. The melting of the butter during rise of the temperature at 5 °C min^−1^ rate exerted three separate peaks, corresponding to low- (LMF), medium- (MMF) and high- (HMF) melting fractions of milk fat in a sequence from lower to higher temperature [49]. The peak melting temperature of the butter was consistent between 21.2 °C to 23.4 °C without showing any apparent difference between Day 0 and 28 (Table 2). On Day 28, melting peaks of Control and WMP showed smaller but significant difference, viz. ~23.3 °C vs. ~21.6 °C, respectively (*p* < 0.05). After phase inversion during the churning process, liquid fat liberated from the ruptured globules forms a continuous phase which during low-temperature storage continues to crystallize, exhibiting a ‘hardening or setting’ of butter [50]. As a result, the SFC was increased in all the samples during four weeks of storage. The increase in crystalline fat, in particular, was the lowest in SC butter. In consequence, the SFC of SC butter remained significantly lower than the control (*p* < 0.05), whereas that of other samples showed minor non-significant variations. Presence of additives are known to act as seeding material influencing the crystallization behavior of fats in dispersed systems [20,51]. The microstructure images showed the arrangement of amphiphilic molecules such as emulsifiers and proteins at the water/oil interface (Figure 1). The apolar groups of the molecule align to the fat phase and polar groups towards the aqueous phase. Hydrophobic emulsifier tail groups protruding in the fat phase act as a template for initiating the heterogeneous interfacial nucleation [20,52]. The interfacial non-fat solids in model water-in-anhydrous milk fat emulsions were also found to enhance the rate of crystallization and crystalline fat content [19]. However, in a complex emulsions like butter, wherein numerous non-fat components are also naturally present, the cumulative effect on fat crystallization becomes comparable at low levels (0.5%, *w*/*w*) of additives [19]. Hence, effects of additives, except SC, on melting temperature and solid fat fraction were found to be inconsistent and insignificant. The distinct effect of SC could be the result of adsorbed portion of SC in intact globular phase wherein hydrophobic groups protruding inside the core cannot be participative with the surrounding fat phase. In fact, the outwardly projecting polar groups of protein on the globule surface could potentially slower the rate of crystallization kinetics due to chemical and structural incompatibility.

#### 3.3.2. Texture and Rheology

The texture analysis performed during the storage Day 1 and 28 showed clear differences in the hardness between the samples and different degrees of effectiveness of the employed additives. The textural attribute and mechanical property of the butter depend in a complex manner on crystal interactions and networking, globular fat portion, and solid/liquid fat fraction. The amount of stress required to fracture the primary interlinkages of the fat crystals and network is linked to the hardness of butter [53,54]. On Day 1, the PGPR and SC butter were softer than the control by 23 and 12%, respectively. The reduction in the hardness and adhesiveness was significant (*p* < 0.05) in PGPR butter. In contrast, WMP and WPI ingredients imparted significantly harder and more adhesive butter texture. The setting of butter during storage elevated the hardness of all the samples, however, at varying magnitude. In control, PGPR, MPC, SC, and Tween 80 butter, 26–38% increase was noticed, whereas, in BMP, WMP, and WPI, it was limited to 10–11% (Table 3). The observed trend in the textural behavior was in poor correlation with the estimated SFC, implying the complexity of structure and potential impact of interaction mechanism between the microstructural elements [7].

Comprehensively, the SC and oil-soluble PGPR emulsifier produced a consistently softer butter in comparison to control, whereas, in the presence of relatively hydrophilic emulsifier Tween 80, hardness of butter reached the maximum. A similar observation was reported by Kapsalis et al. [18] where the use of 2–3% (*w*/*w*) Span 80 emulsifier comprised of dominating hydrophobic moieties improved the softness of butter by 20%, and contrarily, Tween 80 at similar concentration was ineffective. The differences can be associated with the chemical nature and, accordingly, the distribution of additives at oil/water interface in the microstructure, influencing attraction forces and bonding with surrounding crystallites. Both PGPR and Tween 80 are liquid emulsifiers that do not crystallize at refrigeration temperature. Therefore, in the liquid state, the disordered hydrocarbon chains of emulsifier cannot co-solidify or form effective heterogenous templating with surrounding fat phase [17,55], however, could locally enhance the polymorphic evolution of the triglycerides depending on the concentration level [56,57]. Therefore, under applied forces, the weaker interfacial interactions propagate the fracture in the vicinity of droplets inducing an enhanced softness [17] as was noticeable in PGPR butter. The presence of Tween 80, however, did not show similar effectiveness which could be ascribed to differences in the hydrophobic chain structure that may offer a gradual entrapment into growing triglyceride lattice during low-temperature storage [57]. Besides, PGPR butter contained a comparatively higher amount of aqueous phase and so the proportion of liquid (both water and oil) to solid components shifts to more liquid. Hence, relative contribution of solid fat in formation of crystal network reduces implying direct impact on firmness. Similarly, the lower solid fat fraction in SC butter explains the improved softness of butter. On the other side, the harder texture of WPI and WMP butter was accompanied by an increased adhesiveness as compared to control (Table 3). The WPI and WMP butter were also qualitatively gummier than the other samples, which can be attributed to the increased water phase viscosity resulting from dissolved and suspended powder constituents.

The effect of additives on reversible interaction forces in the fat crystal network was characterized through the oscillatory frequency sweep measurements in a linear viscoelastic region [53,58]. The elastic nature of the structure was indicated by the storage modulus (G’) derived through the analysis and was used as a measure of macroscopic consistency, the stiffness of the butter [59]. In this study, observed G’ values were strongly correlated with the hardness parameter, specifically in fresh butter samples (Pearson r = 0.910; *p* = 0.002). In PGPR and SC butter, G’ measures were significantly (*p* < 0.05) lower than the control sample (Table 3). The Tween 80, BMP, and MPC butter showed comparable G’ to that of control butter, whereas WMP and WPI ingredients caused a significant increase in the G’ values. Therefore, the influence of additives on the irreversible and reversible flow of matrix network was of similar trend. However, during the storage, the PGPR butter became 32% stiffer than the control butter, which indicated the dominating effect of developed crystal-fat network and secondary van der Waals interactions under applied linear stress, causing an increase in the stiffness without a similar evolution in the hardness value. On the other side, the effects of SC, WPI, and WMP were observed to be consistent on both hardness and stiffness measures when compared to control butter. Both WPI and WMP solids made butter harder and stiffer than the control and all other ingredients employed in this work.

#### 3.3.3. Tribology

**Figure 3:**
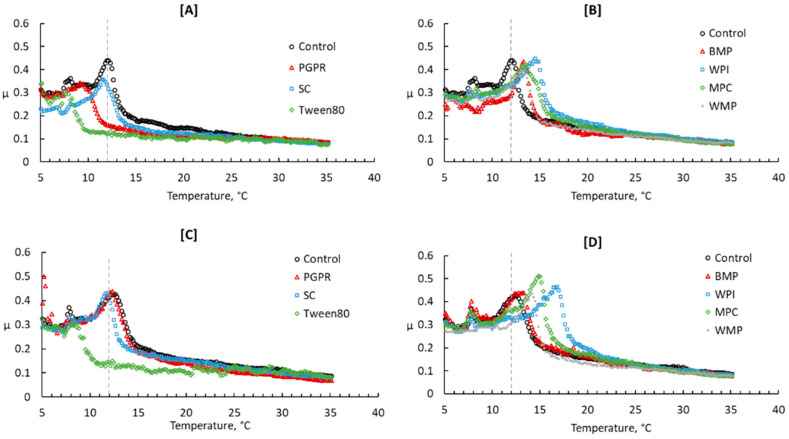
Coefficient of friction of butter samples measured at 15,000 μm s^−1^ of sliding speed and with temperature ramp from 5 °C to 35 °C at 5 °C min^−1^ on Day 1 (**A**,**B**) and 28 (**C**,**D**) of the storage (5 °C). The reference lines show a 12 °C temperature. ‘Control’ sample is without any added additive. PGPR: polyglycerol polyricinoleate, SC: sodium caseinate, BMP: buttermilk powder, WPI: whey protein isolate, MPC: milk protein concentrate, WMP: whole milk powder. Curves of control, Tween 80 and SC are reused from Panchal, et al., 2021 [17]. Copyright 2021 Elsevier.

The tribological analysis of butter samples was carried out to measure the coefficient of friction (µ) parameter. The µ elucidates the quantitative description of surface interactions, the implying friction and lubrication property of the sample [60]. Figure 3 shows the characteristic flow pattern of µ during the temperature ramp. The flow behavior of µ was characterized by the formation of a sharp peak at the initiation of the temperature ramp and then a gradual decline with temperature. At the low temperature, the solid-like surface of the sample exerted more friction with the geometry, as observed as increasing µ to the maximum. After that, the melting of the butter with increasing temperature induced a quick drop in the frictional forces and µ in consequence. Therefore, peak µ values and temperature can be linked to the presence of liquid components and strength of fat crystal network. The employed additives showed a noticeable effect on peak µ values and respective temperatures. On Day 1, the µ of PGPR (0.34 ± 0.02), SC (0.36 ± 0.02), and Tween 80 (0.30 ± 0.05) butter samples reached a maximum in the range of 0.30–0.36, which was distinctly lower than the control butter (0.44 ± 0.03) (Figure 3A). More importantly, the maximum µ of PGPR and Tween 80 butter reached quite early in the temperature ramp (7.8–9.1 °C) compared to that of SC and control butter (11.4–12.1 °C). That means the disruption of the fat crystal interactions and networking was less resistive during the scrapping effect and links to improved flexibility (lubrication) of the solid fat network due to the presence of liquid emulsifiers PGPR and Tween 80. The effect was also reflected in relatively lower G’ values of PGPR, Tween 80 and SC butter, and lower hardness and adhesiveness in PGPR and SC butter (Table 3). However, during storage, the flow pattern and maximum µ values of PGPR and SC butter samples became identical to that of control butter. Whereas the effect of Tween 80 remained consistent (Figure 3C). The presence of other dairy ingredients induced quite a similar flow pattern of µ to that of control butter (Figure 3B); the maximum µ (0.40–0.45) values were observed at a temperature range (13.3–14.5 °C) higher than the control butter. Therefore, an increased resistance to deformation forces was evident, which further enhanced during the storage as maximum µ values increased to 0.44–0.51 between 13.3–16.8 °C (Figure 3D).

## 4. Conclusions

The results of the study highlight the impact of employed additives on the manufacturing, melting properties, and functionality of the butter. The changes observed in mechanical attributes of the butter were illustrative of the ensued modifications in irreversible and reversible interactions in the microstructure in the presence of additives. Comprehensively, PGPR and SC ingredients enhanced the softness of butter with a pronounced effect in the presence of former. Relevantly, the smaller friction coefficient of PGPR and SC butter indicated a more flexible and lubricative microstructure under applied stress. Among other dairy solids, WPI and WMP produced harder, stiffer, and more adhesive butter throughout four weeks of storage, whereas BMP and MPC butter showed similar properties as the control. The observed effects were mainly correlated with the interfacial interactions of additives with surrounding fat phase governed by the chemical and structural compatibility, and impact of dispersed phase rheology.

The outcome signifies the potential of using emulsifiers and dairy-derived ingredients in continuous fat emulsions that could make a noticeable contribution in regulating the overall functionality of the product. Provided that the use of additives did not induce any adverse effect on microstructure and melting temperature, it is possible that selected ingredients could be utilized to tailor the consistency of butter in addition to or bypassing the conventional cream-ageing approach. The concentration level of the additive and addition at a particular stage during processing (before or after churning) would be additional factors that could offer a further scope in product development.

## Figures and Tables

**Table 1:** Churning time and moisture proportion in butter prepared from cream added with different additives at 0.5% (*w*/*w*) level.

Sample	Churning Time, Sec	Moisture, % (*w*/*w*)
Control *	225 ± 21 ^b^	15.47 ± 2.45 ^ab^
PGPR	218 ± 11 ^b^	21.50 ± 1.19 ^a^
Tween 80	160 ± 28 ^b^	15.35 ± 0.28 ^b^
SC	315 ± 21 ^a^	16.89 ± 2.47 ^ab^
WPI	225 ± 21 ^b^	15.17 ± 0.09 ^b^
BMP	195 ± 7 ^b^	14.91 ± 2.04 ^b^
MPC	212 ± 18 ^b^	16.45 ± 1.18 ^ab^
WMP	205 ± 7 ^b^	15.19 ± 0.24 ^b^

* Without any added additive. PGPR: polyglycerol polyricinoleate, SC: sodium caseinate, WPI: whey protein isolate, BMP: buttermilk powder, MPC: milk protein concentrate, WMP: whole milk powder. The shared letter(s) in the respective column indicate(s) no significant difference (*p* < 0.05). Data of control, Tween 80 and SC is reused from Panchal, et al., 2021 [17].

**Table 2:** Peak melting temperature and solid fat content (SFC) of butter samples on Day 1 and 28 of storage (5 °C).

Sample	Peak Melting Temperature (°C)		SFC, %	
	Day 1	Day 28	Day 1	Day 28
Control *	21.8 ± 0.5 ^a^	23.3 ± 0.4 ^a^	42.4 ± 2.4 ^a^	46.3 ± 1.4 ^a^
PGPR	21.2 ± 0.1 ^a^	21.9 ± 0.7 ^ab^	41.3 ± 3.8 ^a^	46.5 ± 0.3 ^a^
Tween 80	23.4 ± 0.1 ^a^	22.9 ± 0.4 ^ab^	38.9 ± 2.1 ^a^	44.2 ± 3.5 ^ab^
SC	23.0 ± 0.2 ^a^	22.7 ± 0.5 ^ab^	37.4 ± 3.3 ^a^	38.3 ± 1.7 ^b^
WPI	22.7 ± 0.2 ^a^	21.9 ± 0.3 ^ab^	38.2 ± 1.5 ^a^	46.1 ± 1.7 ^a^
BMP	21.8 ± 0.6 ^a^	22.1 ± 0.6 ^ab^	42.3 ± 3.7 ^a^	45.4 ± 0.5 ^a^
MPC	22.3 ± 0.1 ^a^	22.2 ± 0.2 ^ab^	44.7 ± 2.6 ^a^	47.1 ± 0.7 ^a^
WMP	22.5 ± 1.4 ^a^	21.6 ± 0.4 ^b^	45.8 ± 0.4 ^a^	50.4 ± 2.1 ^a^

* without any added additive. PGPR: polyglycerol polyricinoleate, SC: sodium caseinate, WPI: whey protein isolate, BMP: buttermilk powder, MPC: milk protein concentrate, WMP: whole milk powder. The shared superscript letter(s) in the respective column and storage day indicate(s) no significant difference (*p* < 0.05). SFC data of control, Tween 80 and SC reused from Panchal, et al., 2021 [17].

**Table 3:** Hardness (N), adhesiveness (N), and elastic modulus (at 400 rad s^−1^) (MPa) of butter samples on Day 1 and 28 during storage (5 °C).

Sample	Hardness, N		Adhesiveness, N		G’, MPa	
	Day 1	Day 28	Day 1	Day 28	Day 1	Day 28
Control *	6.67 ± 0.45 ^bc^	8.44 ± 0.70 ^ab^	1.92 ± 0.10 ^bc^	2.25 ± 0.15 ^ab^	2.62 ± 0.52 ^c^	3.08 ± 0.08 ^cd^
PGPR	5.17 ± 0.28 ^d^	6.58 ± 0.27 ^c^	1.49 ± 0.05 ^d^	1.84 ± 0.10 ^b^	1.45 ± 0.23 ^d^	4.07 ± 0.34 ^abc^
Tween 80	6.72 ± 0.19 ^bc^	9.30 ± 1.10 ^a^	1.97 ± 0.25 ^bc^	2.07 ± 0.34 ^ab^	2.18 ± 0.16 ^cd^	3.47 ± 0.39 ^bcd^
SC	5.88 ± 0.92 ^cd^	7.75 ± 1.07 ^bc^	1.64 ± 0.17 ^cd^	2.50 ± 0.28 ^a^	1.75 ± 0.12 ^d^	2.71 ± 0.80 ^d^
WPI	7.86 ± 0.49 ^a^	8.66 ± 0.46 ^ab^	2.34 ± 0.19 ^a^	2.53 ± 0.27 ^a^	3.96 ± 0.40 ^a^	4.91 ± 0.48 ^ab^
BMP	7.25 ± 0.23 ^ab^	8.05 ± 0.54 ^abc^	2.31 ± 0.15 ^ab^	2.36 ± 0.16 ^ab^	2.47 ± 0.74 ^cd^	3.83 ± 0.53 ^abc^
MPC	6.43 ± 0.13 ^bc^	8.42 ± 0.32 ^ab^	1.93 ± 0.06 ^bc^	2.13 ± 0.10 ^ab^	2.76 ± 0.75 ^bc^	3.92 ± 0.31 ^abc^
WMP	8.08 ± 0.74 ^a^	8.93 ± 0.14 ^ab^	2.48 ± 0.22 ^a^	2.50 ± 0.31 ^a^	3.61 ± 0.57 ^ab^	4.92 ± 0.33 ^a^

* Without any added additive. PGPR: polyglycerol polyricinoleate, SC: sodium caseinate, WPI: whey protein isolate, BMP: buttermilk powder, MPC: milk protein concentrate, WMP: whole milk powder. The shared superscript letter(s) in the respective column and storage day indicate(s) no significant difference (*p* < 0.05). Hardness and G’ data of control, Tween 80 and SC is reused from Panchal, et al., 2021 [17].

## Data Availability

The data presented in this study are available on request from the corresponding author. The data are not publicly available due to privacy restrictions.
